# Mortality after drug-eluting stents vs. coronary artery bypass grafting for left main coronary artery disease: a meta-analysis of randomized controlled trials

**DOI:** 10.1093/eurheartj/ehaa135

**Published:** 2020-03-02

**Authors:** Yousif Ahmad, James P Howard, Ahran D Arnold, Christopher M Cook, Megha Prasad, Ziad A Ali, Manish A Parikh, Ioanna Kosmidou, Darrel P Francis, Jeffrey W Moses, Martin B Leon, Ajay J Kirtane, Gregg W Stone, Dimitri Karmpaliotis

**Affiliations:** Center for Interventional Vascular Therapy, Columbia University Medical Center, NewYork–Presbyterian Hospital, 161 Fort Washington Avenue, New York, NY 10032, USA; National Heart and Lung Institute, Imperial College London, Du Cane Road, London W12 0HS, UK; National Heart and Lung Institute, Imperial College London, Du Cane Road, London W12 0HS, UK; National Heart and Lung Institute, Imperial College London, Du Cane Road, London W12 0HS, UK; National Heart and Lung Institute, Imperial College London, Du Cane Road, London W12 0HS, UK; Center for Interventional Vascular Therapy, Columbia University Medical Center, NewYork–Presbyterian Hospital, 161 Fort Washington Avenue, New York, NY 10032, USA; Center for Interventional Vascular Therapy, Columbia University Medical Center, NewYork–Presbyterian Hospital, 161 Fort Washington Avenue, New York, NY 10032, USA; The Cardiovascular Research Foundation, 1700 Broadway, New York, NY 10019, USA; Center for Interventional Vascular Therapy, Columbia University Medical Center, NewYork–Presbyterian Hospital, 161 Fort Washington Avenue, New York, NY 10032, USA; Center for Interventional Vascular Therapy, Columbia University Medical Center, NewYork–Presbyterian Hospital, 161 Fort Washington Avenue, New York, NY 10032, USA; The Cardiovascular Research Foundation, 1700 Broadway, New York, NY 10019, USA; National Heart and Lung Institute, Imperial College London, Du Cane Road, London W12 0HS, UK; Center for Interventional Vascular Therapy, Columbia University Medical Center, NewYork–Presbyterian Hospital, 161 Fort Washington Avenue, New York, NY 10032, USA; The Cardiovascular Research Foundation, 1700 Broadway, New York, NY 10019, USA; Center for Interventional Vascular Therapy, Columbia University Medical Center, NewYork–Presbyterian Hospital, 161 Fort Washington Avenue, New York, NY 10032, USA; The Cardiovascular Research Foundation, 1700 Broadway, New York, NY 10019, USA; Center for Interventional Vascular Therapy, Columbia University Medical Center, NewYork–Presbyterian Hospital, 161 Fort Washington Avenue, New York, NY 10032, USA; The Cardiovascular Research Foundation, 1700 Broadway, New York, NY 10019, USA; The Cardiovascular Research Foundation, 1700 Broadway, New York, NY 10019, USA; Mount Sinai Hospital, Icahn School of Medicine at Mount Sinai, 1190 Fifth Avenue, New York, NY 10029, USA; Center for Interventional Vascular Therapy, Columbia University Medical Center, NewYork–Presbyterian Hospital, 161 Fort Washington Avenue, New York, NY 10032, USA

**Keywords:** Left main stem, PCI, CABG

## Abstract

**Aims:**

The optimal method of revascularization for patients with left main coronary artery disease (LMCAD) is controversial. Coronary artery bypass graft surgery (CABG) has traditionally been considered the gold standard therapy, and recent randomized trials comparing CABG with percutaneous coronary intervention (PCI) with drug-eluting stents (DES) have reported conflicting outcomes. We, therefore, performed a systematic review and updated meta-analysis comparing CABG to PCI with DES for the treatment of LMCAD.

**Methods and results:**

We systematically identified all randomized trials comparing PCI with DES vs. CABG in patients with LMCAD. The primary efficacy endpoint was all-cause mortality. Secondary endpoints included cardiac death, myocardial infarction (MI), stroke, and unplanned revascularization. All analyses were by intention-to-treat. There were five eligible trials in which 4612 patients were randomized. The weighted mean follow-up duration was 67.1 months. There were no significant differences between PCI and CABG for the risk of all-cause mortality [relative risk (RR) 1.03, 95% confidence interval (CI) 0.81–1.32; *P* = 0.779] or cardiac death (RR 1.03, 95% CI 0.79–1.34; *P* = 0.817). There were also no significant differences in the risk of stroke (RR 0.74, 95% CI 0.35–1.50; *P* = 0.400) or MI (RR 1.22, 95% CI 0.96–1.56; *P* = 0.110). Percutaneous coronary intervention was associated with an increased risk of unplanned revascularization (RR 1.73, 95% CI 1.49–2.02; *P* < 0.001).

**Conclusion:**

The totality of randomized clinical trial evidence demonstrated similar long-term mortality after PCI with DES compared with CABG in patients with LMCAD. Nor were there significant differences in cardiac death, stroke, or MI between PCI and CABG. Unplanned revascularization procedures were less common after CABG compared with PCI. These findings may inform clinical decision-making between cardiologists, surgeons, and patients with LMCAD.

**Figure:**
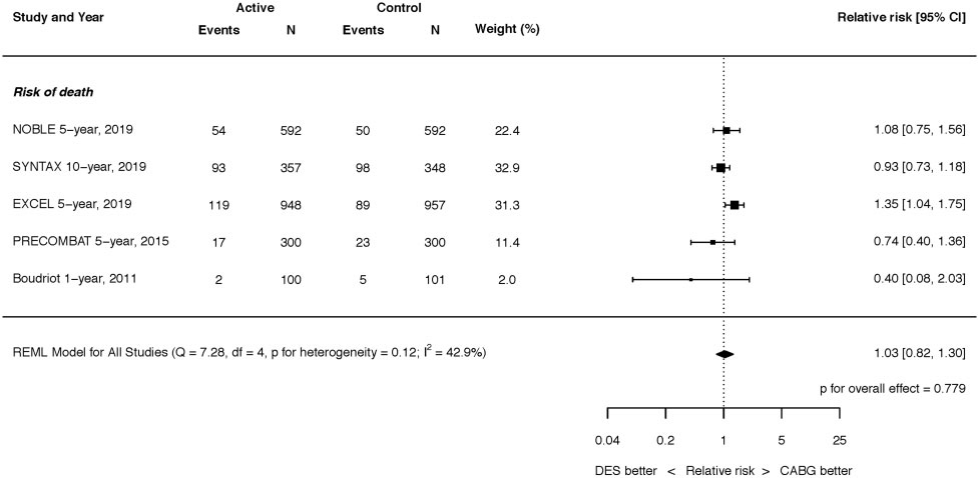

## Introduction

The optimal method of revascularization for patients with left main coronary artery disease (LMCAD) is controversial. Coronary artery bypass graft surgery (CABG) has traditionally been considered the gold standard therapy,^1^ although percutaneous coronary intervention (PCI) is being increasingly performed. Based on the randomized clinical trials (RCTs) comparing CABG and PCI with drug-eluting stents (DES), the 2018 European Guidelines and 2017 US appropriate use criteria recommended PCI as an appropriate alternative to CABG in patients with LMCAD and low-to-intermediate anatomical complexity.^2^ ^,^ ^3^ However, given sample size considerations, all prior trials have relied on composite outcomes as their primary endpoints and were under-powered for important low-frequency endpoints, such as death, stroke, and myocardial infarction (MI). In addition, long-term follow-up (5–10 years) has now been performed for most of these trials. We, therefore, performed a systematic review and up-to-date meta-analysis comparing CABG vs. PCI with DES for the treatment of LMCAD, including for the first time the long-term follow-up from EXCEL, NOBLE, and SYNTAX, and focusing on individual clinical endpoints. 

## Methods

The present analysis was conducted in accordance with published PRISMA guidance^4^ and was prospectively registered at the PROSPERO international prospective register of systematic reviews (ID 163240).

### Search strategy

We performed a systematic search of the MEDLINE, Cochrane Central Register of Controlled Trials, and Embase databases from December 2000 through December 2019 for all trials of LMCAD revascularization. Our search strings included (‘left main stem’ OR ‘left main coronary artery disease’) AND (‘percutaneous coronary intervention’ OR ‘drug-eluting stents’) AND (‘coronary artery bypass grafting’ OR ‘CABG’). We hand-searched the bibliographies of selected studies and meta-analyses to identify further eligible studies. Abstracts were reviewed for suitability and articles accordingly retrieved. Two independent authors performed the search and literature screening (J.H. and A.A.), with disputes resolved by consensus following discussion with a third author (Y.A.).

### Inclusion criteria

Only RCTs were eligible. Trials were eligible if they reported clinical outcome data following randomization to CABG or PCI with DES. Observational and unpublished studies were not eligible.

### Endpoints

The primary efficacy endpoint was all-cause mortality. Secondary endpoints were cardiac (or cardiovascular) death, stroke, MI, and unplanned revascularization. Each trial’s definition of each adverse event was used. Cardiac death was used for the secondary death endpoint unless the trial only reported cardiovascular death. All MI consisted of procedural plus non-procedural MIs, which are also reported separately; both components needed to be adjudicated to be included in the all MI endpoint.

### Data extraction and analysis

Two authors (Y.A. and A.A.) independently abstracted the data from included trials, verified by a third author (J.H.). Included studies were assessed using the Cochrane Risk of Bias tool. Tests for publication bias would only be performed in the event of 10 or more trials being included for analysis.^5^

Outcomes were analysed on an intention-to-treat basis. Random-effects meta-analyses were performed using the restricted maximum likelihood estimator. All outcomes were assessed as relative risks (RRs) at the time of last follow-up available for each trial. We also assessed outcomes at 30 days and 12 months where available. We used the *I* ^2^ statistic to assess heterogeneity.^6^ Low heterogeneity was defined as 0–25%; moderate heterogeneity was defined as 25–50%; and substantial heterogeneity was defined as >50%. Sensitivity analyses were using performed with a fixed-effect model, including only trials with at least 5-year follow-up, and using hazard ratios (HRs) as the outcome measure.^7^ Published HRs at time to last follow-up were available from the SYNTAX, NOBLE, PRECOMBAT, and Boudriot trials. The EXCEL trial reported HRs in the index publication,^8^ and the EXCEL principal investigator (G.W.S.) provided the 5-year HRs for the present analysis.^4^ ^,^ ^9–13^ We also performed a sensitivity analysis excluding each trial in turn for all endpoints.

Mean values are expressed as mean ± standard deviation unless otherwise stated. Statistical significance was set at *P* < 0.05. The statistical programming environment R^14^ with the metafor package^15^ was used for all statistical analyses.

## Results

**Figure 1:**
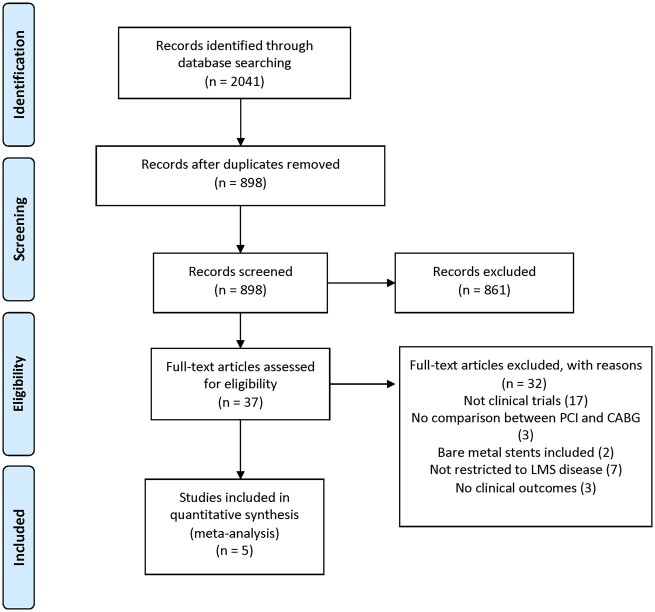
Search strategy and source of included studies.

Five trials^8^ ^,^ ^10–13^ ^,^ ^16–19^ enrolling 4612 patients were eligible (*Figure 1*), including 2303 patients randomized to PCI with DES and 2309 to CABG. Longest follow-up duration was 1 year in one trial,^11^ 5 years in three trials,^10^ ^,^ ^16^ ^,^ ^18^ and 10 years in one trial.^13^ The weighted mean follow-up duration was 67.1 months.

The characteristics of the included trials are summarized in Supplementary material online, *Table S1* and the risk of bias of each trial is shown in Supplementary material online, *Table S2*. The anatomic complexity was on average intermediate according to the SYNTAX score (see Supplementary material online, *Table S1*).

Definitions of outcomes used in each included trial are reported in Supplementary material online, *Table S3*.

### Mortality

**Figure 2:**
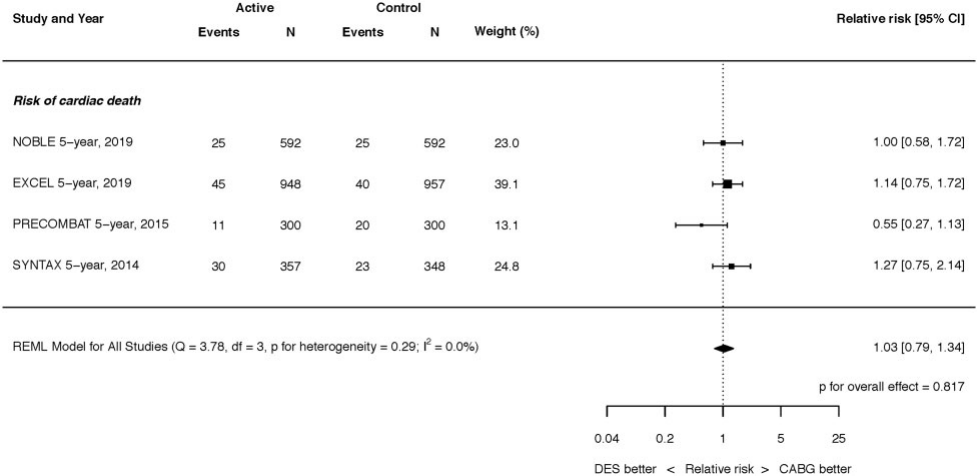
Risk of cardiac death at latest follow-up. Q, Cochran’s Q level of heterogeneity; REML, restricted maximum likelihood; df, degrees of freedom.

At latest follow-up, there was no significant difference in all-cause mortality between PCI with DES vs. CABG [RR 1.03, 95% confidence interval (CI) 0.82–1.30; *P* = 0.779] (*Take home figure*). There was moderate heterogeneity (*I* ^2^ = 42.9%). Cardiac death rates between CABG and PCI with DES were also similar (RR 1.03, 95% CI 0.79–1.34; *P* = 0.817) (*Figure 2*), a finding for which there was no heterogeneity (*I* ^2^ = 0.0%). Nor were there differences in the rates of 30-day or 12-month all-cause or cardiac mortality from those studies in which these data were available (Supplementary material online, *Figures S1–S3*).

**Figure 3:**
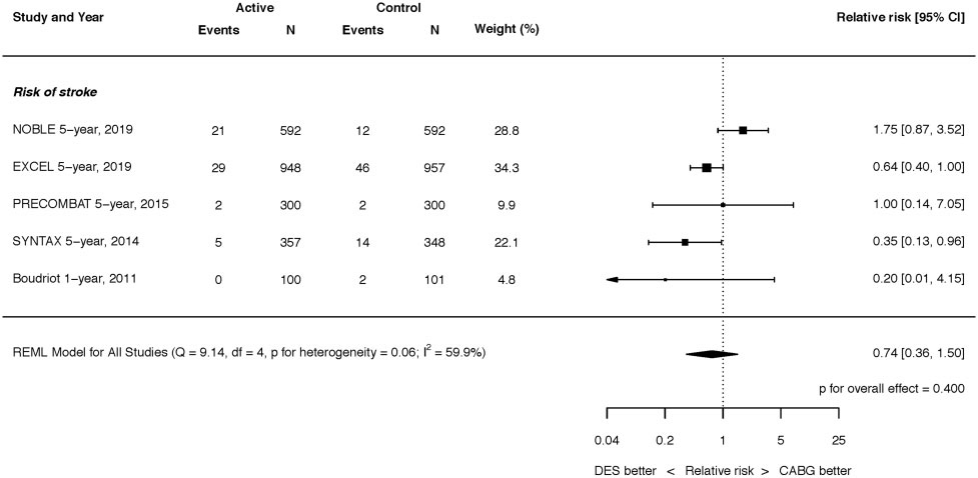
Risk of stroke at latest follow-up. Q, Cochran’s Q level of heterogeneity; REML, restricted maximum likelihood; df, degrees of freedom.

### Stroke

At latest follow-up, there was no statistically significant difference in stroke between PCI with DES vs. CABG (RR 0.74, 95% CI 0.36–1.50; *P* = 0.400), although substantial heterogeneity was present (*I* ^2^ = 59.9%) (*Figure 3*). Two and five trials reported outcomes for stroke at 30 days and 12 months, respectively, the latter finding a lower risk of stroke with PCI (RR 0.38, 95% CI 0.19–0.77; *P* = 0.008) (Supplementary material online, *Figures S4 and S5*).

**Figure 4:**
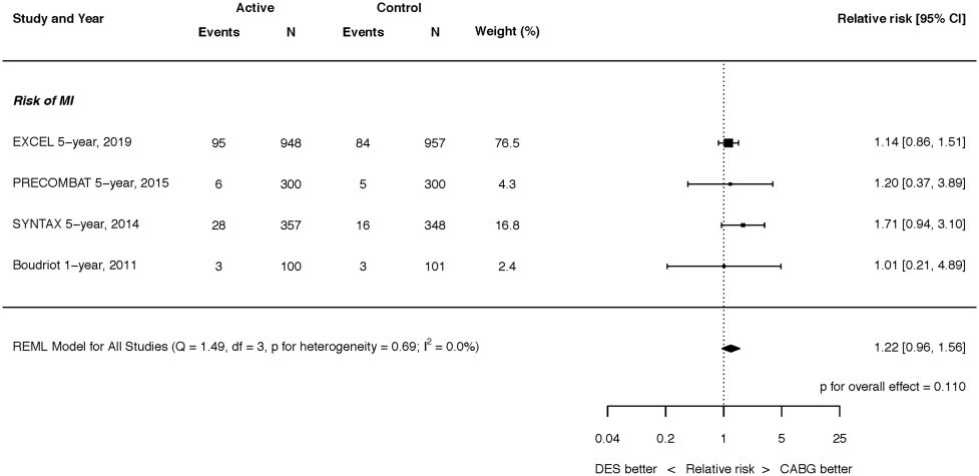
Risk of myocardial infarction at latest follow-up. Q, Cochran’s Q level of heterogeneity; REML, restricted maximum likelihood; df, degrees of freedom.

**Figure 5:**
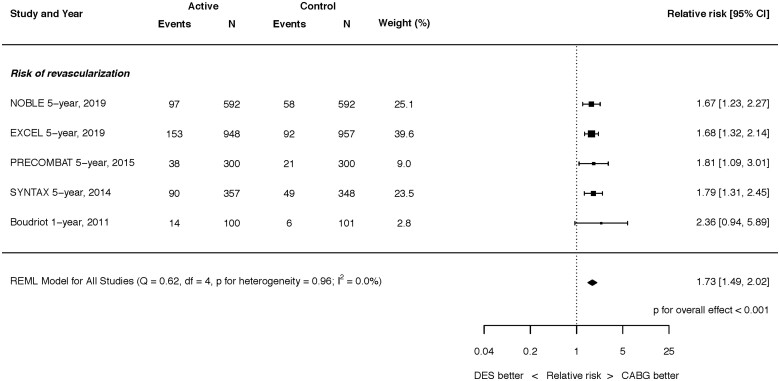
Risk of unplanned revascularization at latest follow-up. Q, Cochran’s Q level of heterogeneity; REML, restricted maximum likelihood; df, degrees of freedom.

### Myocardial infarction

At latest follow-up, there were no significant differences between PCI and CABG in the risks of all MI (RR 1.22, 95% CI 0.96–1.56; *P* = 0.110; *I* ^2^ = 0.0%) (*Figure 4*). Nor was there a significant difference for the 12-month rate of MI between PCI and CABG (Supplementary material online, *Figure S6*). Procedural and non-procedural MI were reported separately in three and two trials, respectively (Supplementary material online, *Figures S7 and S8*). Procedural MI was more common after CABG, whereas non-procedural MI was more common after PCI.

**Take home figure:**
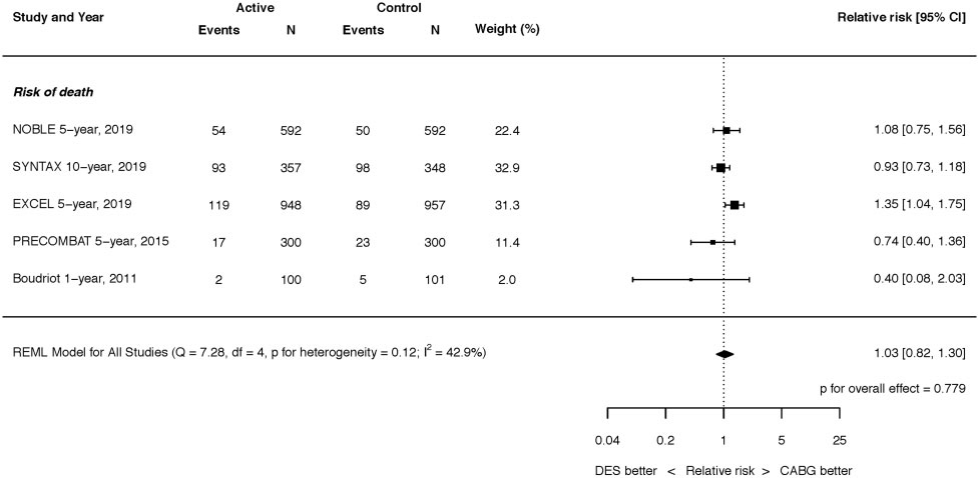
Risk of death at latest follow-up. Q, Cochran’s Q level of heterogeneity; REML, restricted maximum likelihood; df, degrees of freedom.

### Unplanned revascularisation

At latest follow-up, PCI with DES was associated with a higher rate of unplanned revascularization than CABG (RR 1.73, 95% CI 1.49–2.02; *P* < 0.001; *I* ^2^ = 0.0%) (*Figure 5*), a difference which was present by 12 months but not at 30 days (Supplementary material online, *Figures S9 and S10*).

### Sensitivity analyses

The results of the random-effects meta-analyses were consistent for all outcomes when assessed by fixed effects (Supplementary material online, *Figures S11–S15*). The Forest plots for the secondary HR analysis appear in Supplementary material online, *Figures S16–S20* and were consistent with the primary analyses. The outcomes were also consistent when the results were limited to trials with at least 5 years of follow-up (see Supplementary material online, *Figures S21–S25*). The results were also largely similar when each trial was individually excluded (see Supplementary material online, *Figures S26–S49*), with a few notable findings. When the EXCEL trial was removed from the analysis for death, although there was still no significant difference in long-term mortality between the procedures, the moderate heterogeneity between studies that was previously present was no longer observed (RR 0.93, 95% CI 0.77–1.13; *P* = 0.472; *I* ^2^ = 0.0% (Supplementary material online, *Figure 30*). Finally, when the NOBLE trial was excluded, the long-term risk of stroke was reduced after PCI compared with CABG, and the substantial heterogeneity that was previously evident was no longer present (RR 0.58, 95% CI 0.39–0.86, *P* = 0.008; *I* ^2^ = 0.0%; Supplementary material online, *Figure 37*).

## Discussion

The principal finding from the present analysis is that based on the totality of the randomized clinical trial data, at a mean follow-up time of 5.6 years, there was no significant difference in overall mortality after PCI with DES and CABG for the treatment of LMCAD. Similarly, there were no significant long-term differences between PCI and CABG for cardiac death, MI, or stroke. From the available data, the risk of procedural MI was greater with CABG, while the risk for non-procedural MI was greater with PCI (although these endpoints were reported less frequently). Unplanned revascularization was greater with PCI compared with CABG. These results were consistent regardless of the statistical method used (random effects vs. fixed effect and RR vs. HR), and also when limited only to studies with at least 5 years of follow-up. This is the first analysis to incorporate the results of long-term follow-up from the three largest randomized trials of patients with LMCAD (SYNTAX, EXCEL, and NOBLE). These data may importantly inform the heart team and the patient with LMCAD during the clinical decision-making process when selecting between revascularization modalities.

The primary outcome measures of all completed randomized trials comparing PCI and CABG have consisted of composite endpoints of death, MI, and stroke, with or without unplanned revascularization. The use of composite endpoints has been necessary to reduce the required sample size and attendant trial costs but introduces important limitations.^20^ First, the trials differed in the composite endpoints used as primary outcome, leading to different declarations of the principal finding (e.g. inferiority of PCI vs. CABG in trials, such as SYNTAX and NOBLE, in which unplanned revascularization was a component of the composite primary endpoint vs. non-inferiority of PCI vs. CABG in EXCEL in which the primary composite endpoint was death, MI, or stroke without revascularization). Second, the individual components used in the different trials varied somewhat in definition (with the exception of all-cause mortality), the implications of which may be compounded in composite endpoints. Third, each component in a composite endpoint is given equal weighting, meaning a revascularization procedure is rated as equal to death.^21^ In this regard, patients and physicians consistently consider repeat revascularization to be of less importance than death, stroke and MI.^21–23^ Thus, assessment of death, MI, and stroke separately is of particular importance, but all prior studies have been under-powered to examine these individual outcomes with precision.

In particular, the finding of nominally higher 5-year mortality with PCI compared with CABG in the 1905 patient EXCEL trial^16^ has raised uncertainty as to the safety of PCI compared with CABG in LMCAD. However, mortality in this trial was one of >35 under-powered exploratory endpoints for which testing was not adjusted for multiplicity, and the difference was driven by adjudicated non-cardiac deaths due to sepsis and malignancy between 1 and 5 years after the procedure, a mechanism of doubtful aetiologic relationship to the original treatments. Nonetheless, cause of death may be challenging to adjudicate. The present systematic review and meta-analysis was thus performed to examine whether there are true differences in mortality between PCI and CABG for LMCAD. With 4612 randomized patients from five trials, the present study demonstrated similar risk of mortality between PCI and CABG at a mean follow-up of 67.1 months (RR 1.03, 95% CI 0.82–1.30; *P* = 0.779). Of note, however, there was moderate heterogeneity in this result driven by the finding from EXCEL (*I* ^2^ = 42.9%). When the EXCEL trial was excluded, the heterogeneity disappeared (*I* ^2^ = 0%). Finally, if mortality rates were to differ between PCI and CABG, a variance in cardiac mortality would be expected. In this regard, the long-term risks of cardiac death were also similar between PCI and CABG (RR 1.03, 95% CI 0.79–1.34; *P* = 0.817), a finding for which there was no heterogeneity even with the results from EXCEL included.

The similar rates of cardiac death between PCI and CABG in the present study are also consistent with the finding of similar risk of all MI between the procedures (with no heterogeneity between studies). However, compared with PCI, CABG had a higher risk of procedural events, whereas PCI carried a higher risk of late events. Further studies are required to understand the RRs, timing, and causes of MI after PCI and CABG, and the RRs of procedural vs. non-procedural MI.

Patients consider stroke to be as undesirable a complication as death.^22^ ^,^ ^23^ Most prior meta-analyses have reported lower risks of stroke after PCI compared with CABG due to fewer procedural events.^22^ In this study, the 1-year risk of stroke was 62% less after PCI than CABG (RR 0.38, 95% CI 0.19–0.77; *P* = 0.008), although no significant difference in stroke between the procedures was evident at latest follow-up (RR 0.74, 95% CI 0.36–1.50; *P* = 0.400). While similar non-procedural stroke risks during long-term follow-up after PCI and CABG may dilute the RRs over time from an early stroke hazard, substantial heterogeneity between studies was present in the long-term stroke analysis (*I* ^2^ = 59.9%). The source of this heterogeneity was the higher rate of stroke between 1 and 5 years observed in the PCI arm from the NOBLE trial, a finding that to our knowledge has not previously been described in any other study. Absent the NOBLE trial, the long-term risk of stroke was reduced by 42% after PCI compared with CABG (RR 0.58, 95% CI 0.39–0.86; *P* = 0.008), with no heterogeneity between studies (*I* ^2^ = 0%). It has been postulated that prolonged dual antiplatelet therapy after PCI is a possible mechanism for reduced stroke after PCI compared to CABG, but this needs exploration in dedicated studies.

The evolution from balloon angioplasty to bare-metal stents to first and later generation DES has resulted in progressively reduced rates of restenosis and clinically-driven revascularization after PCI.^23–25^ Nonetheless, the present study confirms that even in the DES-era, CABG results in fewer unplanned revascularization procedures than PCI. This finding may be attributed to bypass grafts protecting long segments of mildly or moderately diseased coronary vessels that are likely to progress over time, a mechanism that may also contribute to the lower risk of very late MI after CABG compared with PCI. However, suitable targets for revascularization are less commonly present after CABG than PCI due to accelerated proximal disease progression after bypass, and health status deterioration is substantially greater before and after repeat revascularization after CABG compared with PCI.^26^ Nonetheless, repeat revascularization procedures have been associated with late mortality (although to a lesser extent than stroke and MI).^27–29^ Thus, the difference in long-term revascularization rates after PCI vs. CABG is one factor that should be considered in heart team discussions.

### Limitations

First, study level meta-analysis provides only aggregate outcome data and precludes detailed examination of temporal relationships or subgroup analysis. Insights to the timing of adverse events were gleaned by sensitivity analysis examining 30-day and 1-year events. An individual patient data pooled analysis has been agreed to in principle by the leaders of the four main randomized trials of PCI with DES vs. CABG in which 5-year follow-up data are available, although these results will not be available soon. Second, individual trials used differing definitions of certain endpoints (especially MI), which is a problem common to all meta-analyses. Of note, the NOBLE trial assessed procedural MI events only in a subset of patients (and in a greater proportion of patients after PCI than CABG), introducing selection bias. This subset of patient was included in our analysis of procedural MI shown in Supplementary material online, *Appendix Figure S7*. Nonetheless, these results (numerically fewer procedural MIs after PCI compared with CABG) were consistent with the findings from the EXCEL trial. However, absent assessment in all patients and reporting of a total MI rate (procedural and non-procedural), the MI endpoint from the NOBLE trial could not be included in the all MI analysis. There was a large difference in non-procedural MI in NOBLE in favour of CABG; it is possible that with systematic ascertainment of procedural MI that the overall MI rates would also have been in favour of CABG, but it is not possible to determine this from the available data. Harmonization of endpoint definitions in future studies would facilitate more accurate synthesis of results. Furthermore, the prognostic impact of MI is dependent on the definition used. Third, different stent platforms were used across the differing trials; the extent to which this impacted the results is uncertain. Fourth, it has been suggested that a mortality benefit of CABG may progressively emerge with long-term follow-up. However, in the SYNTAX trial, there were no significant differences in all-cause mortality between PCI and CABG at 10 years (26.1% vs. 26.7%, respectively; HR 0.90, 95% CI 0.68–1.20; *P* = 0.47) and the trajectory of the mortality curves was not diverging over time.^13^ Finally, we only included RCTs. By their nature, they typically randomize only a small fraction of patients. However, when addressing effects of therapy RCTs are the best method of avoiding consistent bias in either direction in the form of both measured and unmeasured confounders.

## Conclusions

From the present updated systematic review and meta-analysis, the totality of randomized clinical trial evidence demonstrated similar long-term mortality after PCI with DES compared with CABG in patients with LMCAD. Nor were there significant aggregate differences in cardiac death, stroke, or MI between PCI and CABG. Unplanned revascularization procedures were less common after CABG compared with PCI. These findings may be valuable in informing clinical decision-making between cardiologists, surgeons, and patients with LMCAD.

## Supplementary Material

ehaa135_Supplementary_Data

## Notes

**See page 3236 for the editorial comment on this article (doi: 10.1093/eurheartj/ehaa318)**
