# Supplementary material for: Mortality after drug-eluting stents vs. coronary artery bypass grafting for left main coronary artery disease: a meta-analysis of randomized controlled trials
Source: Eur Heart J. 2020 Mar 2;41(34):3228–35. doi: 10.1093/eurheartj/ehaa135 (PMC7557472; doi:10.1093/eurheartj/ehaa135)
Supplement: ehaa135_Supplementary_Data [file eurheartj_41_34_3228_s7.docx]

**Supplementary Appendix**

**Mortality after drug-eluting stents versus coronary artery bypass grafting for left main coronary artery disease: A meta-analysis of randomized controlled trials**

Table 1. Characteristics of the included studies. Page 2

Table 2. Risk of bias assessment. Page 3

Table 3. Summary of primary outcome definitions Pages 4-5

Figures 1-2. Risk of death at 30 days and 12 months. Pages 6-7

Figure 3. Risk of cardiac death at 12 months. Page 8

Figures 4-5. Risk of stroke at 30 days and 12 months. Pages 9-10

Figure 6. Risk of MI at 12 months. Pages 11

Figure 7. Risk of procedural myocardial infarction. Page 12

Figure 8. Risk of nonprocedural myocardial infarction at latest follow-up. Page 13

Figures 9-10. Risk of revascularization at 30 days and 12 months. Pages 14-15

Figures 11-15. Fixed effects analyses. Pages 16-20

Figures 16-20. Hazard ratio analyses. Pages 21-25

Figures 21-25. Analyses for trials with at least 5 years of follow-up. Pages 26-30

Figures 26-49. Analyses excluding each trial in turn. Pages 31-54

**Table 1. Characteristics of included studies**

| **Study acronym** | **Author** | **Year** | **Region** | **N** | **Mean Age*** | **Mean SYNTAX score** | **Follow up years**** | **Entry criteria** | **Stent Type** | **Primary outcome§** | **Secondary outcomes§** |
| --- | --- | --- | --- | --- | --- | --- | --- | --- | --- | --- | --- |
| EXCEL | Stone *et al.* | 2019 | Asia, Europe, North America, South America | 1905 | 66.0  (± 9.6) | Site-reported 20.6 in PCI group, 20.5 in CABG group (26.9 and 26.0 core lab assessed) | 5 (median) | ≥70% LMCA visual stenosis, 50-70% stenosis if significant by invasive or non-invasive testing, SYNTAX ≤32.  Silent ischemia, angina, or ACS. | Everolimus-eluting | Composite of all-cause mortality, MI, or stroke at median 3-year follow-up | Primary outcome composite and components, and addition of unplanned revascularisation and graft occlusion/stenosis (+symptomatic), at 3y, 2y, 1y, 6m, 30d and 7d.  Stent thrombosis (definite/probable: acute, subacute, early, late, very late). Baseline complete revascularisation.  Bleeding: 30d, 3y transfusion, TIMI (major/minor) and BARC. |
| SYNTAX | Thijs *et al.* | 2019 | Europe, USA | 705^ | 65.2  (± 9.7) | 29.6 in PCI group and 30.2 in CABG group (core-lab assessed) | 11.2 (IQR 7.7-12.1) | ≥50% LMCA visual stenosis.  Silent ischemia or stable/unstable angina. | Paclitaxel-eluting | Composite of all-cause mortality, stroke, MI or unplanned revascularisation at 1-year follow-up | Primary outcome and individual components, QoL and cost-effectiveness at 5y, 3y, 6m and 1m. |
| PRECOMBAT | Ahn *et al.* | 2015 | South Korea | 600 | 61.8  (± 10.0) | 24.4 in PCI group and 25.8 in CABG group (core-lab assessed) | 5 | ≥50% LMCA visual stenosis.  Silent ischemia, angina, NSTEACS. | Sirolimus-eluting | Composite of all-cause mortality, MI, stroke or ischaemia-driven revascularisation at 1-year follow-up | Primary outcome and components + non-ischaemia driven revascularisation and stent thrombosis/restenosis at 5y, 4y, 3y, 2y, 1y, 6m and 30d. Graft patency and stent/segment luminal loss at 9 month angiogram. |
| NOBLE | Holm *et al.* | 2020 | Europe | 1184 | 66.2  (± 9.9) | 22.5 in PCI group and 22.4 in CABG group (core-lab assessed) | 4.9 (median) | ≥50% LMCA visual stenosis or FFR ≤0.8.  Angina, ACS. | Biolimus-eluting | Composite of all-cause mortality, stroke, non-index treatment-related MI or unplanned revascularization at 5 years or until 275 events | Primary outcome excluding revascularisation, components of primary outcome, definite stent thrombosis / graft occlusion, CCS and NYHA at 275 events, 5y, 4y, 3y, 2y, 1y and 30d |
| NA | Boudriot *et al.* | 2011 | Germany | 200 | 66  (IQR 62-73) | 24.0 in PCI group and 23.0 in CABG group (site-reported) | 1 | ≥50% LMCA visual stenosis.  Angina, silent ischemia. | Sirolimus-eluting | Composite of cardiac death, MI or unplanned revascularisation at 1-0ear follow-up. | Components of primary outcome, all-cause mortality and CCS. |

*Mean age in years (+/- SD); value for stent group provided where values differ between stent and surgery groups and overall value not reported

**Mean +/- SD, where provided, exact follow-up or median ± Interquartile Range (IQR) if mean not reported); value for stent group provided where values differ between stent and surgery groups and overall value not provided. Where multiple publications exist for different follow-up durations, the longest follow-up is provided here.

§ClinicalTrials.gov registration outcomes listed here. Further details of definitions are provided in Table 3 in the supplementary material.

^LMCA stratified sub-study

ACS – Acute Coronary Syndrome, MI – Myocardial Infarction, LMCA – Left Main Coronary Artery, TIMI – Thrombolysis In Myocardial Infarction, BARC – Bleeding Academic Research Consortium, ULN – Upper Limit of Normal, ECG – Electrocardiogram, CCS – Canadian Cardiovascular Society score, NYHA – New York Hospital Association functional class. LTFU – Loss To Follow Up, MACE - Major Adverse Cardiac Events, CT.gov – ClinicalTrials.gov, LTFU – Loss To Follow Up

**Table 2. Risk of bias assessment**

| **Trial** | Random sequence generation | Allocation concealment | Blinding of participants & personnel* | Blinding of outcome assessment* | Incomplete outcome data | Selective reporting | **Overall Quality*** |
| --- | --- | --- | --- | --- | --- | --- | --- |
| **EXCEL** | Low risk  ‘Interactive Voice Response System’ – central allocation | Low Risk Computer generated | High risk  Un-blinded | Unclear Stroke adjudication by blinded neurologist. No mention of blinding for other outcomes, but all outcome definitions pre-specified in detail | Low risk  Over 90% follow up at 5 years with 3.1 percentage points difference between groups. LTFU censoring and imputation. | Unclear  Almost all endpoints in protocol and CT.gov reported except for Universal Definition of Myocardial Infarction (UD-MI) | **High A well conducted open-label trial but the absence of blinded adjudication of clinical events somewhat reduces the quality of this trial with respective to bias-susceptible outcomes.** |
| **SYNTAX** | Low risk  ‘Interactive Voice Response System’ – central allocation. Although an LMCA sub-study without stratification of randomisation for this characteristic, there was balanced recruitment of LMCA stenoses between groups and this was a pre-specified sub-study | Low risk  Computer generated | High risk  Un-blinded | Unclear  Independent clinical endpoints committee but blinding of committee not specified | Low risk  93% follow-up with less than 2 percentage points difference between groups. LTFU censoring at time of LTFU | Low risk  All endpoints on CT.gov reported | **High A well conducted open-label trial but the absence of blinded adjudication of clinical events reduces the quality of this trial with regard to bias-susceptible outcomes** |
| **PRECOMBAT** | Low risk Interactive web-based response system:  computer-generated randomization in permuted block, with the use of sealed envelopes, and stratified  by centre | Low risk Sealed envelopes | High risk  Un-blinded | Low risk  Blinded adjudication committee | Low risk  93% follow-up with less than 2 percentage points difference between groups. LTFU censoring at time of LTFU. | Low risk  All endpoints on CT.gov reported | **High A well conducted open-label trial with blinded adjudication of clinical events. Being an open-label trial its quality is necessarily limited with respect to bias-susceptible outcomes** |
| **NOBLE** | Low risk  Web-based computerised randomisation in permuted blocks stratified by country and centre | High risk Surgeon or cardiologist could overrule assignment if not eligible or if subject refused. | High risk Un-blinded | Unclear  Independent clinical events committee not specified to be blinded | Unclear  >98% follow-up with 6 in stent arm and 11 in surgery arm LTFU but not included in intention-to-treat analysis. Limited reporting of peri-procedural MI. | Low risk  All endpoints on CT.gov reported | **Intermediate An open-label trial with some risk of biased allocation, adjudication and follow-up analysis** |
| **BOUDRIOT** | Low risk  Central allocation via computerised system | Unclear  Not specified | High risk Un-blinded | Low risk  Blinded clinical event committee | Low risk  1 LTFU excluded from analysis (close to 100% follow-up) | Unclear  MACE on CT.gov included cardiac death but manuscript refers to MACE including all-cause mortality. Only all-cause mortality reported. | **Intermediate A well conducted open-label trial with blinded adjudication of clinical events but discrepant reporting of outcomes** |

**Table 3. Summary of primary outcome definitions**

| **Trial** | **Primary Outcome Definition** |
| --- | --- |
| EXCEL | **Myocardial infarction**  Procedural MI was defined as CK-MB >10xULN, or >5xULN with Q waves, angiographic changes or imaging changes within 72 hours of index revascularisation. Spontaneous MI was defined as troponin or CK-MB value > 1x ULN with either ST changes, Q waves, angiographic changes (graft/stent/native severe stenosis) or imaging changes (regional wall motion abnormality or loss of viable myocardium) >72 hours after index revascularisation.  **Stroke**  Stroke was defined as a rapid-onset, new, persistent neurologic deficit attributed to cerebral blood flow obstruction and/or haemorrhage (as determined by a vascular neurologist / stroke specialist). Criteria: 1) new neurological signs/symptoms consistent with stroke, 2) Duration ≥24 hours or < 24 hours with intervention (eg thrombolysis, angioplasty), imaging changes (infarct/haemorrhage) or death due to neurologic deficit, 3) no alternative cause present, 4) neurology / neurosurgical specialist diagnosis using either imaging (CT, MRI or angiography) or lumbar puncture.  ***Ischaemia-driven revascularisation (IDR)***  CABG or PCI to target/non-target vessel/lesion with ≥50% diameter stenosis by QCA and any of the following: 1) positive functional study in IDR-target-lesion territory, 2) ECG changes at rest in IDR-target-vessel territory, 3) typical ischemic symptoms, 4) IVUS MLA ≤4mm2 for non-LMCA or ≤6mm2 for LMCA (if de novo lesion, plaque burden must be ≥60% or FFR ≤ 0.8). However, IDR was not included in the primary outcome. |
| SYNTAX | **Myocardial infarction**  Spontaneous MI was defined as new, abnormal Q waves or enzyme change: >10% of CK-MB/total-CK ratio or CK-MB ≥5x ULN at least 7 days after index revascularisation. Procedural MI was defined as the same within 7 days of the index revascularization  **Stroke**  Acute event related to impairment of cerebral circulation lasting >24 hours resulting in irreversible brain damage or permanent body impairment as evaluated by a local neurologist.  **Repeat revascularisation**  Any revascularisation by CABG or PCI |
| PRECOMBAT | **Myocardial infarction**  Spontaneous MI was defined as typical rise and gradual fall of troponin (or more rapid for CK-MB) >48 hours after index revascularisation with at least one of the following: 1) typical or atypical ischemic symptoms, 2) new Q waves / LBBB, 3) enzyme changes (CK-MB > 1xULN). Procedural MI was defined as new Q waves / LBBB and CK-MB ≥5x ULN within 48 hours of index revascularisation.  **Stroke**  Sudden onset symptoms consistent with stroke due to vascular lesions (haemorrhage, thrombo-embolism) lasting >24 hours  **Ischaemia-driven revascularisation**  CABG or PCI to index lesion with ≥50% diameter stenosis by QCA and any of the following: 1) positive functional study in IDR-target-lesion territory, 2) ECG changes at rest in IDR-target-vessel territory, 3) ischemic symptoms. |
| NOBLE | **Myocardial infarction**  The primary protocol MI definition was spontaneous MI. Spontaneous MI was defined as troponin value >1x ULN with either ischemic symptoms, ST changes or Q waves. angiographic changes (graft/stent/native severe stenosis) or imaging changes (regional wall motion abnormality or loss of viable myocardium). Procedural MI was defined as CK-MB >10xULN, or >5xULN with Q waves, angiographic changes or imaging changes, in the context of a normal baseline CK-MB / troponin. However procedural MI was not included in the primary outcome.  **Stroke**  Ischemic or haemorrhagic stroke verified by CT or MRI.  **Repeat revascularisation**  Any CABG or PCI during follow-up. |
| BOUDRIOT | **Myocardial infarction**  Increase in CK-MB >3x ULN after PCI or >5x ULN after CABG with ECG changes.  **Revascularisation**  Any CABG or PCI during follow-up. |

CABG – Coronary Artery Bypass Grafting, PCI – Percutaneous Catheter Intervention, QCA – Quantitative Coronary Angiography, IDR – Ischemia-driven Revascularisation, MI – Myocardial Infarction, CT – computed Tomography, MRI – Magnetic Resonance Imaging, IVUS – Intra-vascular Ultrasound.

**Figure 1. Risk of death at 30 days**

**Figure 2. Risk of death at 12 months**

**Figure 3. Risk of cardiac death at 12 months**

**Figure 4. Risk of stroke at 30 days**

**Figure 5. Risk of stroke at 12 months**

**Figure 6. Risk of myocardial infarction at 12 months**

**Figure 7. Risk of procedural myocardial infarction***

*Within 72 hours after PCI and CABG in the NOBLE and EXCEL trials and within 30 days post-procedure in the Boudriot et al trial.

**Figure 8. Risk of non-procedural myocardial infarction at latest follow-up**

**Figure 9. Risk of unplanned revascularization at 30 days**

**Figure 10. Risk of unplanned revascularization at 12 months**

**Figure 11. Fixed effect analysis for the risk of death at latest follow-up**

**Figure 12. Fixed effect analysis for the risk of cardiac death at latest follow-up**

**Figure 13. Fixed effect analysis for the risk of stroke at time of latest follow-up**

**Figure 14. Fixed effect analysis for the risk of myocardial infarction at latest follow-up**

**Figure 15. Fixed effect analysis for the risk of unplanned revascularization at latest follow-up**

**Figure 16. Hazard ratio analysis for all-cause mortality at latest follow-up in each study**

**Figure 17. Hazard ratio analysis for cardiac death at latest follow-up in each study**

**Figure 18. Hazard ratio analysis for stroke at latest follow-up in each study**

**Figure 19. Hazard ratio analysis for myocardial infarction at latest follow-up in each study**

**Figure 20. Hazard ratio analysis for unplanned revascularization at latest follow-up in each study**

**Figure 21. Sensitivity analysis including only trials with at least 5-year follow-up for all-cause mortality at latest follow-up**

**Figure 22. Sensitivity analysis including only trials with at least 5-year follow-up for cardiac death at latest follow-up**

**Figure 23. Sensitivity analysis including only trials with at least 5-year follow-up for stroke at latest follow-up**

**Figure 24. Sensitivity analysis including only trials with at least 5-year follow-up for myocardial infarction at latest follow-up**

**Figure 25. Sensitivity analysis including only trials with at least 5-year follow-up for unplanned revascularization at latest follow-up**

**Figure 26. Sensitivity analysis excluding the Boudriot trial for the risk of death at time of latest follow-up**

**Figure 27. Sensitivity analysis excluding the Boudriot trial for the risk of stroke at time of latest follow-up**

**Figure 28. Sensitivity analysis excluding the Boudriot trial for the risk of myocardial infarction at time of latest follow-up**

**Figure 29. Sensitivity analysis excluding the Boudriot trial for the risk of unplanned revascularization at time of latest follow-up**

**Figure 30. Sensitivity analysis excluding the EXCEL trial for the risk of death at time of latest follow-up**

**Figure 31. Sensitivity analysis excluding the EXCEL trial for the risk of cardiac death at time of latest follow-up**

**Figure 32. Sensitivity analysis excluding the EXCEL trial for the risk of stroke at time of latest follow-up**

**Figure 33. Sensitivity analysis excluding the EXCEL trial for the risk of myocardial infarction at time of latest follow-up**

**Figure 34. Sensitivity analysis excluding the EXCEL trial for the risk of unplanned revascularization at time of latest follow-up**

**Figure 35. Sensitivity analysis excluding the NOBLE trial for the risk of death at time of latest follow-up**

**Figure 36. Sensitivity analysis excluding the NOBLE trial for the risk of cardiac death at time of latest follow-up**

**Figure 37. Sensitivity analysis excluding the NOBLE trial for the risk of stroke at time of latest follow-up**

**Figure 38. Sensitivity analysis excluding the NOBLE trial for the risk of myocardial infarction at time of latest follow-up**

**Figure 39. Sensitivity analysis excluding the NOBLE trial for the risk of unplanned revascularization at time of latest follow-up**

**Figure 40. Sensitivity analysis excluding the PRECOMBAT trial for the risk of death at time of latest follow-up**

**Figure 41. Sensitivity analysis excluding the PRECOMBAT trial for the risk of cardiac death at time of latest follow-up**

**Figure 42. Sensitivity analysis excluding the PRECOMBAT trial for the risk of stroke at time of latest follow-up**

**Figure 43. Sensitivity analysis excluding the PRECOMBAT trial for the risk of myocardial infarction at time of latest follow-up**

**Figure 44. Sensitivity analysis excluding the PRECOMBAT trial for the risk of unplanned revascularization at time of latest follow-up**

**Figure 45. Sensitivity analysis excluding the SYNTAX trial for the risk of death at time of latest follow-up**

**Figure 46. Sensitivity analysis excluding the SYNTAX trial for the risk of cardiac death at time of latest follow-up**

**Figure 47. Sensitivity analysis excluding the SYNTAX trial for the risk of stroke at time of latest follow-up**

**Figure 48. Sensitivity analysis excluding the SYNTAX trial for the risk of myocardial infarction at time of latest follow-up**

**Figure 49. Sensitivity analysis excluding the SYNTAX trial for the risk of unplanned revascularization at time of latest follow-up**
